# miR-361-5p Mediates SMAD4 to Promote Porcine Granulosa Cell Apoptosis through VEGFA

**DOI:** 10.3390/biom10091281

**Published:** 2020-09-04

**Authors:** Mengnan Ma, Jinbi Zhang, Xiaomeng Gao, Wang Yao, Qifa Li, Zengxiang Pan

**Affiliations:** College of Animal Science and Technology, Nanjing Agricultural University, Nanjing 210095, China; 18795975502@163.com (M.M.); zhangjinbi@njau.edu.cn (J.Z.); 2016105013@njau.edu.cn (X.G.); njauyw@163.com (W.Y.); liqifa@njau.edu.cn (Q.L.)

**Keywords:** GC apoptosis, VEGFA, miR-361-5p, TGF-β signaling, SMAD4, follicular atresia

## Abstract

Follicular atresia is an inevitable degenerative process that occurs in mammalian ovarian follicles. The molecular events involved in atresia, particularly granulosa cell apoptosis, have long attracted researchers’ attention. Vascular endothelial growth factor A (VEGFA) is downregulated during follicular atresia in porcine ovaries and serves as an inhibitor of apoptosis in granulosa cells. In addition, transforming growth factor (TGF)-βsignaling has been considered a central trigger in granulosa cell apoptosis. However, the link between TGF-β signaling and VEGFA is unknown. We proved that miR-361-5p is significantly upregulated during the atresia process and that it promotes GC apoptosis by directly targeting the *VEGFA* 3′UTR. In addition, we revealed that the miR-361-5p coding gene *MIR361* was significantly downregulated by SMAD4, the central intracellular mediator of TGF-β signaling, that bound to the *MIR361* promoter. In conclusion, our findings expanded what is known about *VEGFA* posttranscriptional regulation and revealed a complete SMAD4/miR-361-5p/VEGFA regulatory network in ovarian granulosa cell apoptosis. These data provide useful references for follicular atresia and ovarian physiological function studies.

## 1. Introduction

Follicular atresia is a common physiological phenomenon that can occur at all stages of follicular development in mammals. At puberty, the porcine primordial follicle reserve contains approximately five million primordial follicles, most of which go through the atresia process when they become larger than 1 mm in diameter, which occurs during the antral stage. The atresia rate remarkably increases in antral follicles of approximately 3–5 mm in diameter [1]. Ultimately, the majority of follicles disappear before maturation, and less than 14% are ovulated [2].

Adequate blood flow is necessary for oxygen and nutrient supply in the ovary and is possibly a rate-limiting step in the selection and maturation of dominant follicles destined for ovulation [3]. The vascular endothelial growth factor (VEGF) family, which is composed of at least six members (VEGF A–F), is involved in the formation of blood vessels. VEGFA was the first VEGF member identified, and it is the most widely studied molecule; it is primarily known to be a factor that promotes angiogenesis and vasculogenesis [4]. VEGFA functions through its major tyrosine kinase receptor VEGFR2 (also known as kinase insert domain receptor, KDR) [5]. The expression and function of VEGFA have been studied in mammalian ovaries during follicular development. In humans, VEGF mRNA and protein are present in the theca layer and in granulosa cells (GCs) during antral follicle stages, which is when the vascular network is actively developing [6]. In bovines, VEGFA mRNA is expressed in both the theca interna and GCs and the VEGF protein level increases as the developmental stages of follicle growth progress [7]. In pigs, the expression levels of two VEGFA isoforms (*VEGF120* and *VEGF164*) in GCs and the levels of two receptors (*VEGFR1* and *VEGFR2*) in theca cells appear to be higher in follicles of medium and large size than they are in small follicles [8]. VEGF production by granulosa cells was also proved to play a role in aortic endothelial cell proliferation, thus drive angiogenesis in the three-dimensional fibrin gel [9,10]. According to our earlier study in pigs, *VEGFA* mRNA levels were significantly decreased in early atretic follicles compared to healthy follicles [11], which implied a potential role of VEGFA during follicular atresia.

Regarding the transcriptional regulation of VEGFA, transcription factors, including hypoxia-inducible factor (HIF) [12], estrogen receptors α and β (ER α and β) [13], signal transducer and activator of transcription 3 (STAT-3), and Wilms tumor 1 (WT1) [14] were identified to regulate VEGFA through direct promoter binding. In recent years, an increasing number of studies have focused on the negative posttranscriptional regulation of VEGFA by miRNAs. Direct binding microRNAs such as miR-26a [15], miR-93 [16], miR-134 [17], miR-195 [18], miR-203 [19], miR-361-5p [20], and miR-503 [21] to the *VEGFA* 3′UTR has been observed in a variety of cells and conditions, mostly in carcinoma. However, despite its key function in the ovary, the posttranscriptional regulation of VEGFA in ovarian follicles, especially during atresia, is still unknown.

It has been reported that the canonical transforming growth factor (TGF)-β signaling pathway is involved in the proliferation and apoptosis of ovarian granulosa cells [22]. In our previous studies, SMAD family member 4 (SMAD4), the terminal regulatory molecule in TGF-β signaling, was identified as an anti-apoptosis factor in granulosa cells [23]. Interestingly, this function of SMAD4 was found to be related to its direct binding to the promoters of a few miRNA coding genes to mediate the negative regulation of miRNA expression [24], which implied a possible regulatory axis involving SMAD4-miRNA-functional genes in GCs. Thus, we performed this study to identify a potential SMAD4/miR-361-5p/VEGFA regulatory axis in porcine GCs and proved that during follicular atresia, miR-361-5p significantly increases and promotes GC apoptosis by directly targeting the *VEGFA* 3′UTR. The expression of the miR-361-5p coding gene *MIR361* is negatively regulated by the SMAD4 binding. The study not only filled the gap in knowledge regarding miRNA-mediated regulation of VEGFA during follicular atresia but also provided evidence for the anti-apoptotic role of SMAD4 through the transcriptional regulation of miRNAs in GCs.

## 2. Materials and Methods

### 2.1. Follicle Collection

Ovaries were obtained from seven-month old unstimulated commercial replacement large white gilts at a local slaughterhouse. The ovaries were quickly washed twice with 75% ethanol and physiologic saline, and then individual antral follicles, approximately 3 to 5 mm in diameter, were dissected using small scissors and fine forceps and then classified as healthy follicles (HFs) and atretic follicles (AFs) according to follicle shape, GC density, and hormone levels [11]. Briefly, HFs are round with a sharp and continuous granulosa cell membrane, fixed, and visible cumulus-oocyte complex (COC), fine capillary vessels, and clear follicular fluid; HFs may have visible COC, but show gaps in membrane granulosa cells, less capillary vessels and turbid follicular fluid. Follicular components were then separated to further confirm the classification by detecting the ratio of progesterone and 17β-estradiol level (P4/E2) and the antral GC density. Follicles with a P4/E2 ratio of <5 were classified as HFs and a ratio of 5 to 20 as AFs. Densities of <250 cells/µL were classified as HFs, 250–1000 cells/µL were classified as AFs. Animal Ethics Committee of Nanjing Agricultural University, Nanjing, Jiangsu, China (SYXK 2017-0027)

### 2.2. Cell Culture and Transfection

Primary GCs were obtained from HFs by extracting via a syringe with a 20-gauge needle. The COC and ovarian tissue were discarded under a stereo microscope. GCs were then cultured with DMEM/F-12 medium (Invitrogen, Carlsbad, CA, USA) containing 10% fetal bovine serum (Invitrogen, Carlsbad, CA, USA), 100 units/mL penicillin and 100 mg/mL streptomycin at 37 °C in 5% CO2. HEK293 cells were maintained in DMEM medium (Sigma, St. Louis, MO, USA) with 10% fetal bovine serum ate the same condition. The *VEGFA* siRNA, Smad4 siRNA, miR-361-5p mimic, and miR-361-5p inhibitor, and their corresponding control oligos were synthesized by GenePharma (Shanghai, China) (Supplementary Table S1). For transfection porcine GCs were culture for12 h, then transfected with the appropriate plasmids or oligos using Lipofectamine 2000 and Opti-MEM (Invitrogen, Carlsbad, CA, USA), according to the manufacturer’s protocol.

### 2.3. Immunohistochemical Assay

To examine the expression and location of VEGFA in healthy and atretic follicles, immunohistochemical staining was performed according to our previous description [25]. Rabbit polyclonal VEGFA (diluted 1:200 in PBS containing 1% (*w*/*v*) bovine serum albumin, ab9570, Abcam, Cambridge, MA, USA) and a secondary antibody (G1210-2-A, Servicebio, Wuhan, China) were incubated with the slides. Specific protein immunoreactivity was visualized by incubation with 0.05% 3,30-diaminobenzidine (DAB, G1211, Servicebio, Wuhan, China) for 15 min, and the slides were counterstained with haematoxylin (G1004, Servicebio, Wuhan, China). Images were captured under a microscope (Nikon Eclipse E200, Tokyo, Japan).

### 2.4. RNA Extraction and qRT-PCR

Total RNA was extracted from follicles and GCs using TRIzol reagent (Invitrogen, Carlsbad, CA, USA). Then, the extracted total RNA was reverse transcribed to generate cDNA using a Super M-MLV RTase Synthesis Kit, and qRT-PCR was performed using SYBR Premix Ex Taq (Takara, Dalian, China) on an ABI StepOne system (Applied Biosystems, Carlsbad, CA, USA), according to the manufacturer’s instructions. GAPDH was used as an internal control. For miRNA detection, first-strand cDNA synthesis and qRT-PCR were performed using miRNA two-step qRT-PCR SuperMix (TransGen, Beijing, China). U6 was used as an internal control. For each gene primer set, a control containing no cDNA was included, and each reaction was repeated three times for every sample. The primers for qRT-PCR are listed in Supplementary Table S2.

### 2.5. Fluorescent In Situ Hybridization (FISH)

A FAM-labelled probe (5′-GTACCCCTGGAGATTCTGATAA-3′) was specifically synthesized for miR-361-5p, and DAPI was used to stain the cell nuclei. GCs were cultured on coverslips, fixed in 4% paraformaldehyde (containing DEPC) for 20 min, washed while shaking with PBS (pH 7.4) three times, and proteinase K (20 µg/mL) was finally added for 5 min for digestion. Then, all procedures were conducted according to the manufacturer’s instructions (Sevicebio, Wuhan, China). Finally, the images were acquired on a Nikon upright fluorescence microscope (Nikon DS-U3, Tokyo, Japan). Each experiment was performed three times.

### 2.6. Protein Extraction and Western Immunoblotting Analysis

GCs were washed with cold PBS and lysed with RIPA buffer containing 1% phosphatase inhibitor (*v*/*v*) (Beyotime, Shanghai, China) and proteinase inhibitor (Sigma, St. Louis, MO, USA). The protein concentration was determined with a BCA Protein Assay Kit (Beyotime, Shanghai, China), and samples were diluted to the same concentration using 5× Protein Loading Dye (Sangon, Shanghai, China). Total protein extracts were separated by SDS-PAGE on 12% gels. The proteins were then transferred onto PVDF membranes (Millipore, Billerica, MA, USA), and the membranes were blocked with 5% non-fat milk for 2 h. After washing with Tris-buffered saline with Tween (TBST) for 15 s, the membranes were incubated overnight at 4 °C with anti-VEGFA (diluted 1:5000, ab9570, Abcam, Cambridge, MA, USA), anti-Tubulin (diluted 1:1000, 10094-1-AP ProteinTech, Nanjing, China), and anti-CASP3 (diluted 1:1000, 19677-1-AP, ProteinTech, Nanjing, China). Then, the cells were incubated with a secondary peroxidase-conjugated antibody (diluted 1:2000, Cell Signaling Technology, Beverly, MA, USA) for 1 h at room temperature. Chemiluminescence was detected by WesternBright™ ECL (Advansta, Menlo Park, CA, USA) and analyzed using the ImageJ software (Version 1.51w). Each experiment was performed three times.

### 2.7. Plasmid Construction

*VEGFA* 3′UTR fragments containing putative target sites for miR-361-5p and the promoter fragments of the miR-361-5p coding gene (*MIR361*) containing putative SMAD4 binding sites were amplified from porcine genomic DNA and were verified by sequencing. The *VEGFA* 3′UTR fragment was then digested with Nhel and Xbal (Thermo, Waltham, MA, USA) and cloned into a pmirGLO Dual-Luciferase miRNA Target Expression Vector (Promega Corporation, Madison, WI, USA). The *MIR361* promoter fragment was digested with NheI and SacI, and then cloned into a pGL-3 reporter vector (Promega, Madison, WI, USA). The miR-361-5p plasmids with a mutated putative binding site were generated by the ClonExpress Entry One Step Cloning Kit (Vazyme, Nanjing, China), according to the manufacturer’s protocol. Successful mutations were confirmed by sequencing. The overexpression plasmid pcDNA3.1-SMAD4 was generated previously by our group [26]. The primers used here are detailed in Supplementary Table S3.

### 2.8. Luciferase Reporter Assays

After a transfection period of 24 h, the cells and lysates were collected. A Dual-Luciferase Reporter Assay System (Promega Corporation, Madison, WI, USA) was used to quantify luciferase activities following the manufacturer’s instructions. Firefly luciferase activity was normalized to Renilla luciferase activity. Each experiment was performed six times.

### 2.9. Apoptosis Assay

GC apoptosis was measured with an annexin V-FITC/PI staining assay (Vazyme, Nanjing, China) according to the manufacturer’s protocol. A cell-counting machine (Becton Dickinson, Franklin Lakes, NJ, USA) was used for the detection of apoptotic cells based on the principle of fluorescence-activated cell sorting (FACS). The data were analyzed using the FlowJo v7.6 software (Stanford University, Stanford, CA, USA).

### 2.10. Statistical Analysis

All data are presented as the means ± S.E.M. The Prism 5 software (GraphPad Software) was used to perform the statistical analysis. Two-tailed Student’s *t*-tests were used to evaluate the significance when two groups were compared. When three or more groups were compared, a one-way analysis of variance test was performed, and Tukey’s test was used to determine significance between groups. *p*-values of <0.05 and 0.01 were considered to indicate significant and extremely significant differences, respectively.

## 3. Results

### 3.1. VEGFA Is Downregulated in Atretic Follicles

**Figure 1:**
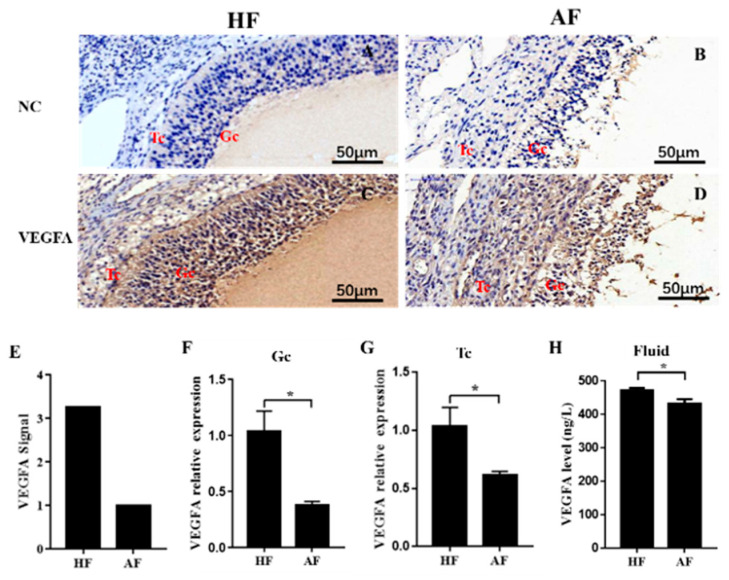
Expression of VEGFA in healthy and atretic antral follicles. (**A**–**D**): Immunolocalization of VEGFA in healthy (**A**,**C**) and atretic (**B**,**D**) antral follicles; €: The signal intensity of VEGFA in follicles detected by the GeneChip Porcine Genome Array; (**F**,**G**): relative expression levels of *VEGFA* in GC and TC, respectively, detected by qRT-PCR; (**H**): expression level of VEGFA in the follicle fluid detected by ELISA. NC, negative control; HF, healthy follicle; AF, atretic follicle; GC, granulosa cell; Tc, theca cell; scale bar = 50 μm. Data are expressed as the mean ± SEM. * *p* < 0.05.

To investigate the VEGFA level during follicular atresia, we first determined the location of VEGFA in antral follicles by immunohistochemistry. The results showed a positive reaction in both theca and granulosa cells, and the brown staining was stronger in HFs (GCs closely arranged) than in AFs (GCs loosely arranged and partially dropped into the follicular cavity) (Figure 1A–D). In addition, mRNA levels of *VEGFA* detected in the whole follicle, GCs, and theca cells (TCs) by GeneChip Porcine GenomeArray (detailed data are contained in reference [11]) or qRT-PCR also suggested a significantly higher expression in HFs than in AFs (Figure 1E–G). ELISA for VEGFA showed a slight but significant decrease in VEGFA content in follicular fluid throughout the atresia process (Figure 1H). These results suggested that VEGFA decreases during porcine follicular atresia.

### 3.2. miR-361-5p Is Upregulated in Atretic Follicles

**Figure 2:**
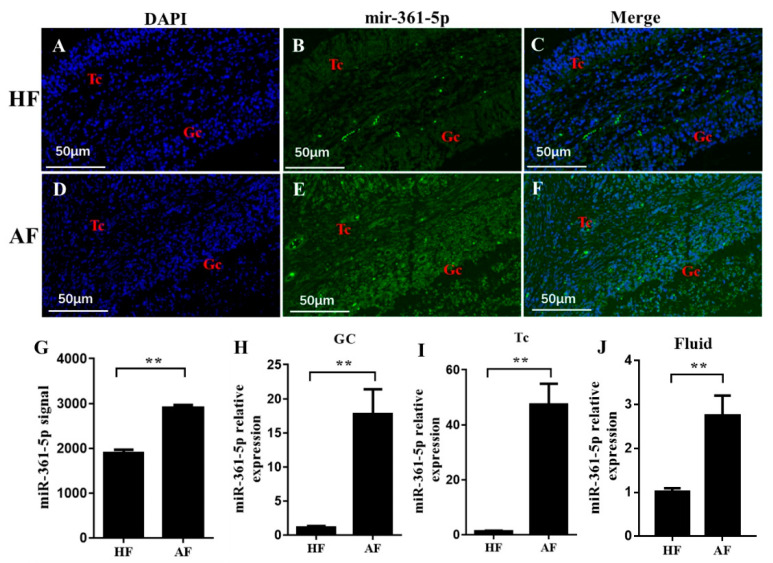
Expression of the mir-361-5p in healthy and atretic antral follicles. (**A**–**F**): RNA-FISH was utilized to examine the localization of mir-361-5p in healthy and atretic antral follicles; (**G**): Signal intensity of mir-361-5p in follicles detected by µParaflo™ microfluidic chip; (**H**–**J**): relative expression levels of mir-361-5p in GC, TC, and follicle fluid, respectively, detected by qRT-PCR. HF, healthy follicle; AF, atretic follicle; GC, granulosa cell; TC, theca cell; scale bar = 50 μm. Data are expressed as the mean ± SEM. ** *p* < 0.01.

To investigate the possible function of miR-361-5p during follicle atresia, we detected its location and expression levels in healthy and atretic follicles. FISH results showed that miR-361-5p was distributed in both TCs and GCs, and the signal was stronger in atretic follicles than in healthy follicles (Figure 2A–F). Further qualitative measurements were collected from the whole follicle, GCs, TCs, and follicular fluid by the microchip (data extracted from the µParaflo™ microfluidic chip used in our previous study for miRNA expression profiles in healthy and atretic follicles [27]) or qRT-PCR, respectively; those results also suggested significantly higher expression of mir-361-5p in atretic follicles than in healthy follicles in each of the follicle compartments (Figure 2G–J). These results implied that miR-361-5p was involved in the atresia process and may be involved in the posttranscriptional regulation of functional genes during follicular atresia.

### 3.3. miR-361-5p Regulates VEGFA by Directly Binding to Its 3′UTR

**Figure 3:**
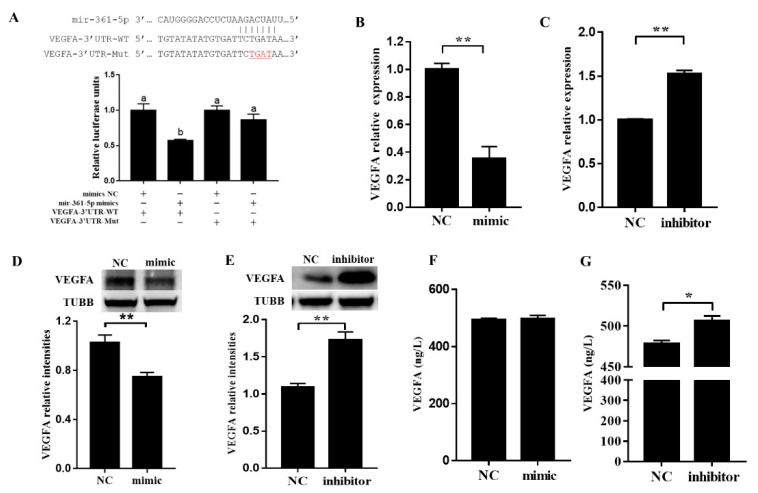
Negative regulation of *VEGFA* by miR-361-5p through direct 3′UTR binding. (**A**): The direct targeting of *VEGFA* by miR-361-5p confirmed by luciferase reporter assay in 293 cells (plus (+) indicates addition of oligos, minus (−) indicates no addition of oligos); (**B**,**C**): The mRNA levels of *VEGFA* after transfection of miR-361-5p mimics or inhibitors in porcine GCs; (**D**,**E**): The protein levels of VEGFA after transfection of miR-361-5p mimics or inhibitor in porcine GCs; (**F**,**G**): The levels of VEGFA after transfection of miR-361-5p mimics and inhibitors in culture media. *n* = 3 cell culture wells per group. Data are expressed as the mean ± SEM. Significant differences (*p* < 0.05) are indicated by different letters or * *p* < 0.05, ** *p* < 0.01.

To further investigate the possible function of miR-361-5p in the regulation of VEGFA expression, the direct targeting of *VEGFA* by miR-361-5p was first predicted by bioinformatic methods and was confirmed by the luciferase reporter assay (Figure 3A). Next, we cultured porcine GCs transfected with miR-361-5p mimics or an inhibitor and then detected the mRNA/protein levels of VEGFA both in GCs and in the culture media. The results showed that both mRNAs (Figure 3B,C), and protein (Figure 3D,E) levels of VEGFA were significantly decreased after transfection with miR-361-5p mimics but increased after transfection with a miR-361-5p inhibitor (Figure 3B–E). In addition, we noticed that the levels of secreted VEGFA in culture media were slightly increased after transfection with a miR-361-5p inhibitor (Figure 3F,G). These results suggested that miR-361-5p negatively affected *VEGFA* expression by directly binding to its 3′UTR in porcine GCs.

### 3.4. miR-361-5p Regulates GC Apoptosis through VEGFA

**Figure 4:**
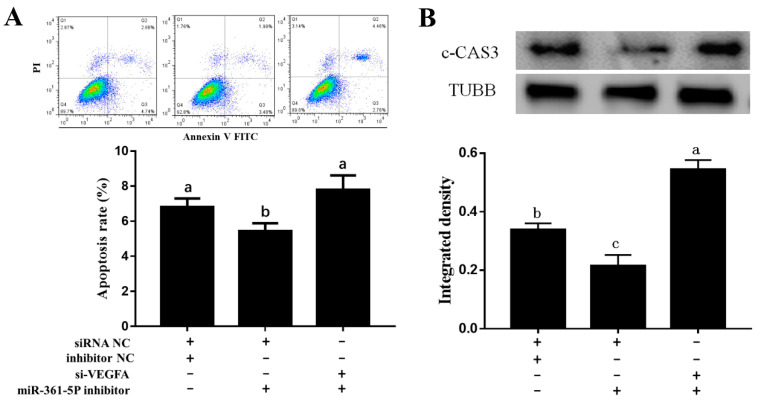
miR-361-5p regulates GC apoptosis through VEGFA. (**A**): GC apoptosis rate decreased after the transfection of miR-361-5p inhibitor and reversed after the addition of *VEGFA* siRNA detected by FACS analysis. (**B**): The protein levels of cleaved caspase 3 (c-CAS3) was down-regulated after the transfection of the miR-361-5p inhibitor and reversed after the addition of *VEGFA* siRNA. Plus (+) indicates addition of siRNAs or inhibitors, minus (−) indicates no addition of siRNAs or inhibitors. Data are expressed as the mean ± SEM. Significant differences (*p* < 0.05) are indicated by different letters.

To determine whether miR-361-5p affects apoptosis of porcine GCs via regulation of VEGFA, we co-transfected a miR-361-5p inhibitor with a VEGFA siRNA. The FACS results suggested that the apoptosis rate was significantly decreased after miR-361-5p inhibitor transfection but reversed after the addition of VEGFA siRNA (Figure 4A). The protein levels of active cleaved Caspase 3 (c-CAS3) also showed a similar pattern (Figure 4B). Thus, miR-361-5p can promote GC apoptosis through VEGFA.

### 3.5. SMAD4 Involved in miR-361-5p-Mediated VEGFA Expression

**Figure 5:**
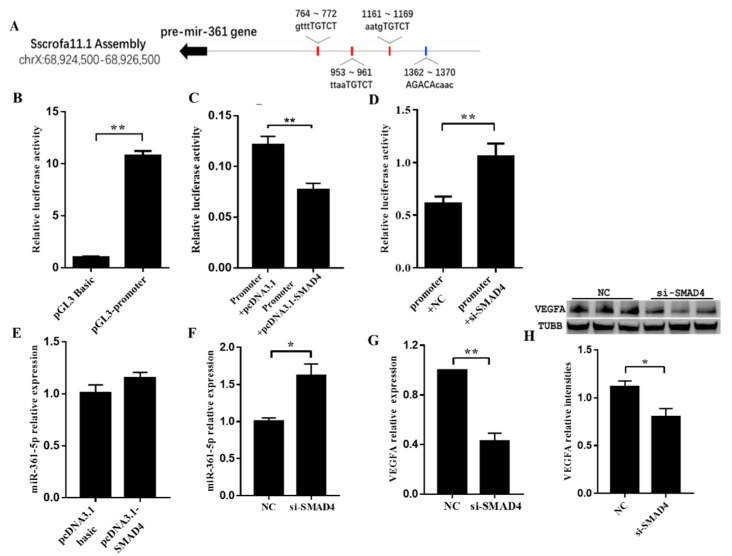
Transcription factor SMAD4 was involved in miR-361-5p mediated VEGFA expression. (**A**): Schematic diagram showing the genome location of the miR-361-5p coding gene and potential SMAD4 binding sites. (**B**): The promoter activity of *MIR361* upstream region confirmed by the dual-luciferase reporter assay; (**C**,**D**): Overexpression and knockdown of SMAD4 weaken and enhanced *MIR361* promoter activity, respectively; (**E**): The expression of miR-361-5p was not affected after SMAD4 overexpression; (**F**): The expression of miR-361-5p was up-regulated after SMAD4 knockdown; (**G**,**H**): The expression of VEGFA mRNA and protein levels were down-regulated after SMAD4 knockdown. *n* = 3 cell culture wells per group. Data are expressed as the mean ± SEM. * *p* < 0.05, ** *p* < 0.01.

To examine whether the expression of miR-361-5p was controlled by the TGF-β signaling pathway, we analyzed the promoter region of the miR-361-5p coding gene *MIR361* for potential binding sites. We identified four SMAD-binding elements (SBEs) within the region (Figure 5A). Next, we confirmed the role of the SBEs in *MIR361* promoter activity with using a dual-luciferase reporter assay (Figure 5B). To further investigate the effect of SMAD4 on the *MIR361* promoter, the increase and knockdown of SMAD4 were achieved by transfecting cells with a SMAD4 overexpression plasmid and siRNA, respectively. The luciferase reporter assay results suggested that SMAD4 had a negative effect on the *MIR361* promoter (Figure 5C,D). In addition, in cultured porcine GCs, overexpression of SMAD4 did not show an apparent effect on miR-361-5p, but knockdown of SMAD4 significantly enhanced miR-361-5p expression (Figure 5E,F). Finally, both qRT-PCR and WB data suggested that knockdown of SMAD4 resulted in a significant decrease in VEGFA expression (Figure 5G,H). These results indicated that SMAD4 functioned as a trans-acting element to negatively regulate *MIR361* transcription, and thereby, adjust VEGFA expression.

## 4. Discussion

**Figure 6:**
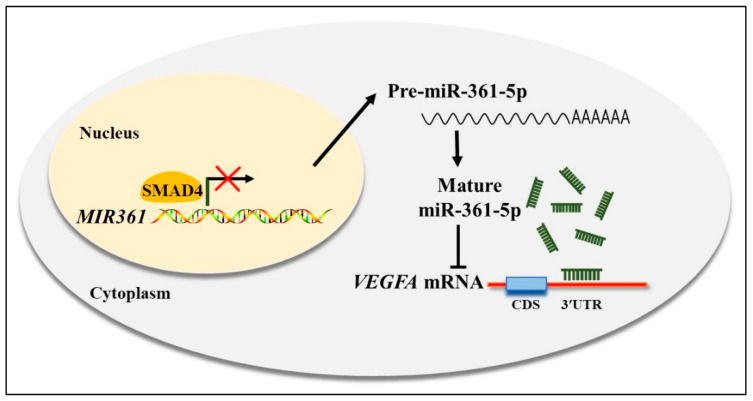
Schematic diagram of SMAD4/miR-361-5p/VEGFA regulatory signaling in porcine GCs. SMAD4 negatively regulates *MIR361* transcription by binding to the *MIR361* promoter, while matured miR-361-5p reduces VEGFA expression level by directly binding to 3′UTR *VEGFA* mRNA.

miRNAs regulate gene expression by binding to specific sequences in target mRNAs, resulting in transcriptional repression [28] or degradation [29] of target mRNAs. In the field of reproduction, the critical roles of miRNAs in ovarian function, follicle development, and luteal formation have continually attracted attention and were recently connected to GC apoptosis and follicular atresia processes in humans, mice, bovines and pigs [30]. Our previous study, which compared the differential expression of miRNAs in healthy and atretic follicles, suggested a significant increase in miR-361-5p during atresia [27]. The fact that miR-361-5p was found to inhibit cell proliferation and metabolism and induce apoptosis in many cancer studies [31,32,33] also implied a possible relationship between miR-361-5p and GC apoptosis during the atresia process. In this study, we confirmed a direct interaction between miR-361-5p and VEGFA, which is mainly produced in GCs, and plays essential roles in angiogenesis, GC function, and oocyte development in antral follicles [25]. In addition, our study explored the transcriptional regulation of the miR-361-5p coding gene *MIR361* by SMAD4 and identified a straightforward regulatory network of SMAD4/miR-361-5p/VEGFA. Briefly, miR-361-5p reduces *VEGFA* mRNA expression by direct binding, thus promoting GC apoptosis. Further, SMAD4 increases VEGFA levels through negative regulation of *MIR361* expression through promoter binding at the transcription level Figure 6.

It is well known that the expression of VEGFA could be regulated at the transcriptional level by several cis-acting mechanisms and factors such as HIF [12], ER α, β [13], STAT-3, and WT1 [14]. The direct regulation of VEGFA by TGF-β signaling was reported to be carried out by SMAD3 binding. For example, in vascular smooth muscle cells, TGF-β led to the formation of a complex of SMAD3 and HIF-1α that, in turn, activated the VEGFA transcription [34]. SMAD4, on the other hand, is a universal mediator that plays a role in canonical TGF-β signal transduction into the nucleus, where SMAD4 complexes regulate gene transcription positively or negatively with different coactivator or corepressor factors [35].

In porcine ovarian granulosa cells, the participation of TGF-β signaling has been proved by SMAD4 knockdown [36]. Since then, the apoptosis-inducing capacity of SMAD4 was gradually revealed. Some studies suggested that SMAD4 affects the FSH response because knockdown of SMAD4 significantly inhibited FSH-induced GC proliferation and estradiol production [37]. Additionally, SMAD4 was proven to downregulate miR-143 expression by binding to its promoter, thus opposing the GC apoptosis caused by miR-143 targeting of FSHR [24]. Interestingly, instead of direct binding, the regulatory function of SMAD4 in ovarian granulosa cells is more achieved on the posttranscriptional level through non-coding RNAs, and SMAD4 generally serves as a negative regulator, which resists miRNA transcription, in this case [38]. Our results provide further evidence that SMAD4 could reduce VEGFA levels through miRNA-mediated mechanisms, which adds knowledge to the specific function of TGF-β signaling in granulosa cells. The preferences of SMAD4 in miRNA and gene binding and their particularity in different cell types awaits further investigation.

## 5. Conclusions

Our data provide direct evidence that miR-361-5p is upregulated during follicular atresia and that it enhances GC apoptosis by directly targeting the 3′UTR of *VEGFA* mRNA and downregulating its expression. In addition, TGF-β signaling might play a part in VEGFA-mediated GC apoptosis by transcriptional regulation of miR-361-5p expression via its common mediator SMAD4. Overall, our findings broaden the knowledge of VEGFA posttranscriptional regulation in ovarian GC apoptosis and provide novel insights into the mechanism underlying follicular atresia and ovarian physiological function in mammalian ovaries.

## Supplementary Materials

The following are available online at https://www.mdpi.com/2218-273X/10/9/1281/s1, Table S1: specific small inference RNA sequences and microRNA sequence are as following, Table S2: qRT-PCR primers were as following, Table S3: Plasmid construction primers are as following.
