# Supplementary material for: miR-361-5p Mediates SMAD4 to Promote Porcine Granulosa Cell Apoptosis through VEGFA
Source: Biomolecules. 2020 Sep 4;10(9):1281. doi: 10.3390/biom10091281 (PMC7563248; doi:10.3390/biom10091281)
Supplement: Supplementary file 1 [file biomolecules-10-01281-s001.pdf]

Table S1: specific small inference RNA sequences and microRNA sequence are as following.

Gene	sequence (5’→3’)
Negative control	Sense:UUCUCCGAACGUGUCACGUTT<br>Anti-sense:ACGUGACACGUUCGGAGAATT
VEGFA siRNA	Sense:GCGAGGCAGCUUGAGUUAATT<br>Anti-sense:UUAACUCAAGCUGCCUCGCTT
Negative control	Sense:UUCUCCGAACGUGUCACGUTT<br>Anti-sense:ACGUGACACGUUCGGAGAATT
Smad4 siRNA	Sense:CACCAGGAAUUGAUCUCUCAGGAUU<br>Anti-sense:AAUCCUGAGAGAUCAAUUCCUGGUG
MiR-361-5p mimic NC	UUGUACUACACAAAAGUACUG
MiR-361-5p mimic	UUAUCAGAAUCUCCAGGGGUAC
MiR-361-5p inhibitor NC	CAGUACUUUUGUGUAGUACAA
MiR-361-5p inhibitor	GUACCCCUGGAGAUUCUGAUAA

Table S2: qRT-PCR primers were as following:

Gene	Accession No.	Primer sequence (5’→3’)	Size (bp)	Annealing temperature (°C)
VEGFA	XM_013977975.1	F: ATTTCTTGAAGGTAAAGATGCA<br>R: CAGCCCCACAGAGGGTCTCA	117	60
KDR	XM_003128987.6	F: CCCAGTGAGACTCTGTGCG<br>R: CAGGTGTCATAGCGGAAGAAC	275	60
GAPDH	AF017079	F: CGTGTCGGTTGTGGATCT<br>R: CTCAGTGTAGCCCAGGAT	120	60
BCL2	AB271960	F: TTCTTTGAGTTCGGTGGGG<br>R: CCAGGAGAAATCAAATAGAGGC	195	60

BAX	AJ606301	F: GATGCCTTTGTGGAGCTGTATG<br>R: CCCGTGGACTTCACTTATGG	154	60
Smad4	NM214072	F:ATTGGTGTTCCATTGCCTAC<br>R:TGGTCACTAAGGCACCTGAC	250	60
MiR-361-5p		F:CGCGCTAGCAGCACGTAAAT<br>R:GTGCAGGGTCCGAGGT		60
Pre-miR-361-5p	NR029848.1	F:CAGGGGTACTTATAATTTGAAAAAG<br>R:AGGAGGAGGAAGCAAATCA	72	60
U6		F:CGCTTCGGCAGCACATATAC<br>R:TTCACGAATTTGCGTGTCAT		60
MiR-361-5p		GTCGTATCCAGTGCAGGGTCCGAGGTATTCGCACTGGATACGACCGCCAAT		Stem-loop structure for RT

Table S3: Plasmid construction primers are as following

Gene	Primer sequence (5'→3')	Size (bp)	Annealing temperature (°C)
VEGFA	F: ATTTCTTGAAGGTAAAGATGCA<br>R: CAGCCCCACAGAGGGTCTCA	184	56
MiR361 promoter	F:GAGCTCTAACAAGAGTCTAGGGCAGTG<br>R:CTCGAGATGGTTCCCTGCTCTAATC	182	61
PcDNA3.0-Smad4	F:ATGGACAATATGTCTATTAC<br>R:TCAGTCTAAAGGCTGTG	1948	58
